# Supplementary material for: A long-term study on zooplankton in two contrasting cascade reservoirs (Iguaçu River, Brazil): effects of inter-annual, seasonal, and environmental factors
Source: PeerJ. 2020 May 5;8:e8979. doi: 10.7717/peerj.8979 (PMC7207214; doi:10.7717/peerj.8979)
Supplement: Table S1 [file peerj-08-8979-s003.docx]

**Supplemental information**

**Table S1** **Results (*P*-values) of the four-way ANOVA for the environmental variables.**

|  | Environmental variables | | | | | | | | | | | |
| --- | --- | --- | --- | --- | --- | --- | --- | --- | --- | --- | --- | --- |
| Effects | Chl *a* | DO | Z_max_ | Z_SD_ | Cond | pH | TDS | TN | TP | Turb | WT | TSI |
| Year | **<0.001** | **<0.001** | **<0.001** | **<0.001** | **<0.001** | **<0.001** | **<0.001** | **<0.001** | **<0.001** | **<0.001** | **0.012** | **<0.001** |
| Period | 0.559 | **<0.001** | 0.363 | 0.473 | 0.526 | 0.221 | 0.623 | 0.149 | **0.027** | 0.279 | **<0.001** | 0.131 |
| Reservoir | **0.027** | 0.406 | **<0.001** | **0.002** | 0.544 | 0.757 | 0.171 | 0.716 | 0.656 | 0.368 | 0.225 | **0.003** |
| Environment | **0.047** | **0.001** | **<0.001** | **<0.001** | 0.394 | **0.008** | 0.461 | 0.609 | 0.914 | **<0.001** | **<0.001** | **<0.001** |
| Year*Period | **<0.001** | **0.015** | **0.024** | **<0.001** | **<0.001** | **<0.001** | **<0.001** | **0.006** | **<0.001** | **<0.001** | 0.129 | **<0.001** |
| Year*Reservoir | 0.658 | 0.901 | **0.034** | 0.956 | 0.657 | 0.864 | 0.77 | 0.958 | 0.237 | 0.992 | 0.997 | 0.654 |
| Period*Reservoir | 0.876 | **0.036** | 0.322 | 0.214 | 0.382 | 0.43 | 0.434 | **0.039** | 0.424 | 0.45 | 0.755 | 0.412 |
| Year*Environment | 0.979 | 0.862 | **0.021** | 0.989 | 1 | 0.992 | 1 | 1 | 1 | 1 | 1 | 0.999 |
| Period*Environment | 0.456 | **0.009** | 0.629 | 0.59 | 0.964 | 0.062 | 0.85 | 0.595 | 0.985 | 0.493 | 0.051 | 0.362 |
| Reservoir*Environment | 0.632 | 0.619 | **<0.001** | **<0.001** | 0.993 | 0.437 | 0.943 | 0.953 | 0.938 | **0.037** | 0.446 | **0.002** |
| Year*Period*Reservoir | 0.283 | 0.779 | 0.315 | 0.983 | 0.975 | 0.457 | 0.506 | 0.986 | 0.968 | 0.903 | 0.995 | 0.702 |
| Year*Period*Environment | 0.999 | 0.996 | 0.678 | 1 | 1 | 1 | 0.863 | 1 | 1 | 1 | 1 | 1 |
| Year*Reservoir*Environment | 0.967 | 0.924 | 0.229 | 0.984 | 1 | 0.957 | 0.982 | 1 | 1 | 1 | 1 | 0.996 |
| Period*Reservoir*Environment | 0.914 | 0.557 | 0.873 | 0.249 | 0.864 | 0.74 | 0.436 | 0.938 | 0.977 | 0.835 | 0.552 | 0.478 |
| Year*Period*Reservoir*Environment | 0.953 | 0.996 | 0.251 | 1 | 1 | 0.999 | 0.997 | 1 | 1 | 1 | 1 | 1 |

**Notes:** Year indicates the sampling years (2003–18); period refers to winter and summer; reservoir refers to Salto Santiago and Salto Osório reservoirs; and environment refers to the four environments (fluvial, transitional, lacustrine, and downstream). Significant differences (*P* < 0.05) are shown in bold; asterisks indicate an interaction between the effects.
