# Supplementary material for: A long-term study on zooplankton in two contrasting cascade reservoirs (Iguaçu River, Brazil): effects of inter-annual, seasonal, and environmental factors
Source: PeerJ. 2020 May 5;8:e8979. doi: 10.7717/peerj.8979 (PMC7207214; doi:10.7717/peerj.8979)
Supplement: Table S2 [file peerj-08-8979-s004.docx]

**Supplemental information**

**Table S2** **Results of the ANOSIM test showing zooplankton community differences between the sampling years for the Salto Santiago and Salto Osório Reservoirs.**

| Salto Santiago | | | | | | | | | | | | | | | | |
| --- | --- | --- | --- | --- | --- | --- | --- | --- | --- | --- | --- | --- | --- | --- | --- | --- |
| Years | 2003 | 2004 | 2005 | 2006 | 2007 | 2008 | 2009 | 2010 | 2011 | 2012 | 2013 | 2014 | 2015 | 2016 | 2017 | 2018 |
| 2003 | − | 1 | 1 | 1 | 0.516 | 1 | 0.432 | 0.168 | **0.024** | 0.06 | **0.036** | 0.828 | 1 | 0.396 | 0.9 | 0.876 |
| 2004 |  | − | 1 | 1 | 1 | 1 | 0.264 | 1 | **0.024** | 0.06 | **0.036** | 0.588 | 0.216 | **0.048** | 0.24 | 0.24 |
| 2005 |  |  | − | 1 | 1 | 1 | 1 | 0.324 | **0.024** | **0.036** | **0.024** | 0.132 | **0.012** | **0.012** | **0.048** | 0.06 |
| 2006 |  |  |  | − | 1 | 1 | 1 | 1 | 0.144 | 0.072 | **0.024** | 0.252 | **0.024** | 0.072 | 0.072 | **0.036** |
| 2007 |  |  |  |  | − | 1 | **0.024** | 1 | 0.12 | **0.024** | **0.024** | 0.132 | **0.048** | **0.024** | **0.024** | **0.024** |
| 2008 |  |  |  |  |  | − | 1 | 1 | 0.144 | **0.048** | **0.012** | 1 | 0.96 | 0.852 | 0.408 | 1 |
| 2009 |  |  |  |  |  |  | − | 0.408 | **0.036** | 0.06 | **0.036** | 0.06 | 0.084 | **0.048** | 0.06 | **0.024** |
| 2010 |  |  |  |  |  |  |  | − | 1 | 0.876 | 1 | 1 | 0.384 | 0.804 | 0.78 | 1 |
| 2011 |  |  |  |  |  |  |  |  | − | 1 | 0.948 | 0.66 | **0.024** | 0.096 | **0.024** | 0.48 |
| 2012 |  |  |  |  |  |  |  |  |  | − | 1 | 0.168 | **0.012** | **0.036** | **0.012** | 0.204 |
| 2013 |  |  |  |  |  |  |  |  |  |  | − | 1 | 0.06 | 0.108 | 0.228 | 0.96 |
| 2014 |  |  |  |  |  |  |  |  |  |  |  | − | 1 | 1 | 1 | 1 |
| 2015 |  |  |  |  |  |  |  |  |  |  |  |  | − | 1 | 1 | 1 |
| 2016 |  |  |  |  |  |  |  |  |  |  |  |  |  | − | 1 | 1 |
| 2017 |  |  |  |  |  |  |  |  |  |  |  |  |  |  | − | 1 |
| 2018 |  |  |  |  |  |  |  |  |  |  |  |  |  |  |  | − |
| Salto Osório | | | | | | | | | | | | | | | | |
| Years | 2003 | 2004 | 2005 | 2006 | 2007 | 2008 | 2009 | 2010 | 2011 | 2012 | 2013 | 2014 | 2015 | 2016 | 2017 | 2018 |
| 2003 | − | 1 | 0.6 | 0.528 | 0.552 | 0.276 | 0.06 | 0.12 | **0.012** | 0.072 | **0.036** | **0.024** | 0.096 | 0.072 | **0.012** | **0.024** |
| 2004 |  | − | 1 | 1 | 1 | 1 | 0.12 | 0.228 | **0.024** | **0.036** | **0.012** | **0.036** | 0.108 | **0.024** | **0.024** | **0.036** |
| 2005 |  |  | − | 1 | 1 | 1 | 1 | 0.252 | **0.024** | **0.024** | **0.024** | **0.036** | **0.048** | 0.06 | **0.024** | **0.012** |
| 2006 |  |  |  | − | 1 | 1 | 0.492 | 1 | **0.024** | **0.024** | **0.012** | **0.048** | 0.084 | 0.132 | **0.048** | 0.132 |
| 2007 |  |  |  |  | − | 1 | 0.336 | 1 | 0.18 | **0.036** | **0.036** | 0.264 | 1 | 0.876 | 0.084 | 0.552 |
| 2008 |  |  |  |  |  | − | 1 | 1 | 0.132 | **0.024** | 0.096 | 0.276 | 1 | 0.828 | 0.264 | 1 |
| 2009 |  |  |  |  |  |  | − | 0.072 | **0.024** | **0.024** | **0.012** | **0.036** | 0.072 | **0.012** | **0.024** | **0.012** |
| 2010 |  |  |  |  |  |  |  | − | 1 | 0.096 | 0.06 | 0.348 | 1 | 0.192 | 0.084 | 1 |
| 2011 |  |  |  |  |  |  |  |  | − | 1 | 1 | 1 | 1 | 1 | 0.204 | 1 |
| 2012 |  |  |  |  |  |  |  |  |  | − | 1 | **0.036** | 0.324 | **0.048** | 0.156 | 1 |
| 2013 |  |  |  |  |  |  |  |  |  |  | − | 1 | 0.984 | 0.216 | 0.48 | 1 |
| 2014 |  |  |  |  |  |  |  |  |  |  |  | − | 1 | 1 | 1 | 1 |
| 2015 |  |  |  |  |  |  |  |  |  |  |  |  | − | 1 | 1 | 1 |
| 2016 |  |  |  |  |  |  |  |  |  |  |  |  |  | − | 1 | 1 |
| 2017 |  |  |  |  |  |  |  |  |  |  |  |  |  |  | − | 1 |
| 2018 |  |  |  |  |  |  |  |  |  |  |  |  |  |  |  | − |

**Notes:** Bold font indicates significant differences (*P* < 0.05).
